# Few amino acid signatures distinguish HIV-1 subtype B pandemic and non-pandemic strains

**DOI:** 10.1371/journal.pone.0238995

**Published:** 2020-09-22

**Authors:** Ighor Arantes, Marcelo Ribeiro-Alves, Suwellen S. D. de Azevedo, Edson Delatorre, Gonzalo Bello

**Affiliations:** 1 Fundação Oswaldo Cruz, Instituto Oswaldo Cruz, Laboratório de AIDS & Imunologia Molecular, Rio de Janeiro, Brazil; 2 Fundação Oswaldo Cruz, Instituto Nacional de Infectologia Evandro Chagas, Laboratório de Pesquisa Clínica em DST-AIDS, Rio de Janeiro, Brazil; 3 Universidade Federal do Espírito Santo, Departamento de Biologia, Centro de Ciências Exatas, Naturais e da Saúde, Alegre, Brazil; Instituto de Salud Carlos III, SPAIN

## Abstract

The Human Immunodeficiency Virus Type I (HIV-1) subtype B comprises approximately 10% of all HIV infections in the world. The HIV-1 subtype B epidemic comprehends a pandemic variant (named B_PANDEMIC_) disseminated worldwide and non-pandemic variants (named B_CAR_) that are mostly restricted to the Caribbean. The goal of this work was the identification of amino acid signatures (AAs) characteristic to the B_CAR_ and B_PANDEMIC_ variants. To this end, we analyzed HIV-1 subtype B full-length (n = 486) and partial (n = 814) genomic sequences from the Americas classified within the B_CAR_ and B_PANDEMIC_ clades and reconstructed the sequences of their most recent common ancestors (MRCA). Analysis of contemporary HIV-1 sequences revealed 13 AAs between B_CAR_ and B_PANDEMIC_ variants (four on Gag, three on Pol, three on Rev, and one in Vif, Vpu, and Tat) of which only two (one on Gag and one on Pol) were traced to the MRCA. All AAs correspond to polymorphic sites located outside essential functional proteins domains, except the AAs in Tat. The absence of stringent AAs inherited from their ancestors between modern B_CAR_ and B_PANDEMIC_ variants support that ecological factors, rather than viral determinants, were the main driving force behind the successful spread of the B_PANDEMIC_ strain.

## Introduction

The Human Immunodeficiency Virus Type I (HIV-1) is one of the most important human pathogens that have emerged in the 20^th^ century and exhibits an extraordinary degree of genetic variability, organized in groups (M, N, O, and P), subtypes (A-D, F-H, and J-L), sub-subtypes, and many recombinant forms [1]. HIV-1 subtype B comprises approximately 10% of all HIV infections in the world, being one of the most globally disseminated HIV-1 variants and the most prevalent subtype in the Americas, Europe, Oceania, as well as some Asian countries [2].

The HIV-1 subtype B shares a common ancestor with subtype D that was present in Kinshasa, capital of Democratic Republic of Congo (DRC), by the early 1940s [3]. The currently accepted hypothesis about the emergence and worldwide spread of subtype B is punctuated by two major founder events. The first event occurred when the HIV-1 subtype B ancestor strain moved from the DRC into the Caribbean around the middle 1960s, establishing the first HIV epidemic outside the African region [4–7]. The second major event took place when one subtype B strain spread from the Caribbean to the U.S. around the late 1960s and got access to high-risk transmission networks globally connected that fueled the establishment of a pandemic clade (B_PANDEMIC_) responsible for most of the infections of this subtype worldwide [8, 9]. In contrast, other non-pandemic subtype B lineages (B_CAR_) spread and circulates at high prevalence in several Caribbean islands and the Northern South American region, but were not successfully disseminated worldwide [4, 6, 8–11].

The introduction of the B_PANDEMIC_ ancestor in highly connected transmission networks in the U.S. during the very early phase of the epidemic probably accounts for the successful dissemination of this viral variant worldwide [4, 12, 13]. Differences in viral fitness, however, may also have shaped the uneven geographic distribution of distinct HIV-1 subtype B lineages. Viral transmissibility correlated with the plasma viremia during chronic infection [14], and some studies found that plasma viremia within subtype B is highly heritable, thus indicating that this trait depends strongly on the virus genotype [15–18]. Notably, a significant trend for higher viral loads among subjects infected with B_PANDEMIC_ relative to B_CAR_ strains was recently described in French Guiana [11] which may have played a role in the differential transmissibility of the two viral strains. Studies of molecular signatures in non-pandemic subtype B lineages, however, have been limited so far to the analysis of the *env* gene of B_CAR_ strains circulating in Trinidad and Tobago [19, 20].

The objective of this work is to identify amino acid signatures (AAs) that can distinguish B_CAR_ and B_PANDEMIC_ strains by the analysis of full-length (FL) and partial genome subtype B sequences representative of different Caribbean islands and American countries and by reconstructing the sequences of the most recent common ancestors (MRCA) of B_CAR_ and B_PANDEMIC_ strains. These analyses may provide crucial information to understand the potential relevance of viral genetic determinants on the epidemic spread of different subtype B variants.

## Materials and methods

### HIV-1 subtypes B and D datasets

HIV-1 subtype B FL genome sequences from North America (n = 330), South America (n = 151), and the Caribbean (n = 25), as well as Sub-Saharan African subtype D FL genome sequences (n = 18) that were available at Los Alamos HIV Database (http://www.hiv.lanl.gov) by November 2018, were downloaded (S1 and S2 Tables). We also downloaded HIV-1 subtype B sequences from Caribbean islands with high prevalence of B_CAR_ strains and that covered selected genomic regions of *gag* (HXB2 coordinates: 1,264 to 2,148), *pol* (HXB2 coordinates: 2,253 to 3,272), and *env* (HXB2 coordinates: 6,450 to 8,480) and HIV-1 *pol* sequences (HXB2 coordinates: 2,253 to 3,272) from drug-naïve individuals of Caribbean origin (S1 Table). Only one sequence for patient was selected.

### Clade assignment of HIV-1 subtype B sequences

The HIV-1 subtype B sequences were aligned with the HIV Align online tool [21] and then manually curated. The presence of putative intra-subtype recombinant sequences was evaluated using the RDP4 software [22], being deemed as recombinant those sequences selected as such by three or more of the algorithms. The remaining FL and partial subtype B genome sequences were classified as either B_CAR_ or B_PANDEMIC_ based on their placement on a maximum likelihood (ML) phylogenetic tree inferred with the PhyML program [23] under the best nucleotide substitution model, selected using an online web server [24]. The heuristic tree search was performed using the SPR branch-swapping algorithm, and the reliability of the obtained topology was estimated with the approximate likelihood-ratio test [25] based on the Shimodaira–Hasegawa-like procedure. The ML phylogenetic trees were visualized using the FigTree v1.4.4 program [26].

### Reconstruction of ancestral subtype B sequences

To reduce computation time while retaining most viral diversity information, we generate a “non-redundant” subtype B FL genome subset by removing very closely related B_PANDEMIC_ sequences. To achieve this goal, B_PANDEMIC_ sequences with identity above 91.5% were grouped with the CD-HIT program [27] using an online web server [28], and only one sequence per cluster (the oldest one) was selected. To map amino acid changes fixed during the evolution of subtype B, FL genome sequences of the MRCA of B_CAR_ and B_PANDEMIC_ strains were then reconstructed using a Bayesian Markov Chain Monte Carlo (MCMC) approach, as implemented in BEAST v1.8 [29, 30] with BEAGLE [31] to improve run-time. The Bayesian time-tree was reconstructed using the GTR+I+Г4 nucleotide substitution model [32], a relaxed uncorrelated lognormal molecular clock model [33], and a Bayesian Skyline coalescent tree prior [34] with non-informative default priors. MCMC chains were run for 100 × 10^6^ generations, and convergence and uncertainty of parameter estimates were assessed by calculating the Effective Sample Size (ESS) and 95% Highest Probability Density (HPD) values, respectively, after excluding the initial 10% of each run with Tracer v1.7 [35]. The convergence of parameters was considered when ESS ≥ 200. After the exclusion of sequences corresponding to the burn-in phase, the remaining ones were utilized to generate an FL consensus sequence for each MRCA using the Seaview version 4 program [36].

### Amino acid signature (AAs) analyses

Nucleotide sequences were translated, and the software VESPA (Viral signature pattern analysis) [37] was used to identify positions in which the most common amino acid differs between B_CAR_ and B_PANDEMIC_ datasets as well as between subtype B and subtype D datasets. These positions were then selected, and for each a Chi-square test, as implement in R version 3.5.0 [38], was used to evaluate the statistical significance of their different amino acidic compositions. AAs were defined by positions in which both the most common amino acid was different, and the overall amino acid composition was significantly different between viral clades. For specific genomic regions corresponding to the structural *gag*, *pol*, and *env* genes, the number of B_CAR_ sequences was expanded, and the process to identify AAs between B_CAR_ and B_PANDEMIC_ datasets previously detailed was applied. The false discovery rate (FDR) method was used to correct for multiple hypothesis testing and to reduce false positives. Statistical significance was defined as FDR < 0.05.

### Phenotypic prediction

We determine the frequency of genetic polymorphisms in accessory (Vif, Vpr, Nef) and regulatory (Rev) HIV-1 proteins of B_PANDEMIC_ and FL/expanded B_CAR_ datasets that were previously associated with slow HIV-1 disease progression and differential function [39–50]. The Geno2Pheno algorithm was used to predict the chemokine receptor tropism of the B_PANDEMIC_ and expanded B_CAR_ *env* dataset sequences based on their V3 region [51]. V3 was studied through the 11/25 rule (R or K at position 11 and/or K at position 25) [52–54], and the combination of positively charged amino acids at position 25 and an increase in total net charge [55]. The frequency of surveillance drug-resistance mutations (SDRMs) was also explored in B_CAR_ and B_PANDEMIC_ *pol* sequences retrieved from drug-naïve individuals of Caribbean origin using the Calibrated Population Resistance (CPR) tool (http://cpr.stanford.edu/cpr.cgi) [56]. A Chi-square test, as implement in R version 3.5.0 [38] was used to evaluate the significance of the results in both cases. Statistical significance was defined as *p*-values < 0.05.

## Results

### Classification of HIV-1 B_CAR_ and B_PANDEMIC_ FL sequences

**Fig 1:**
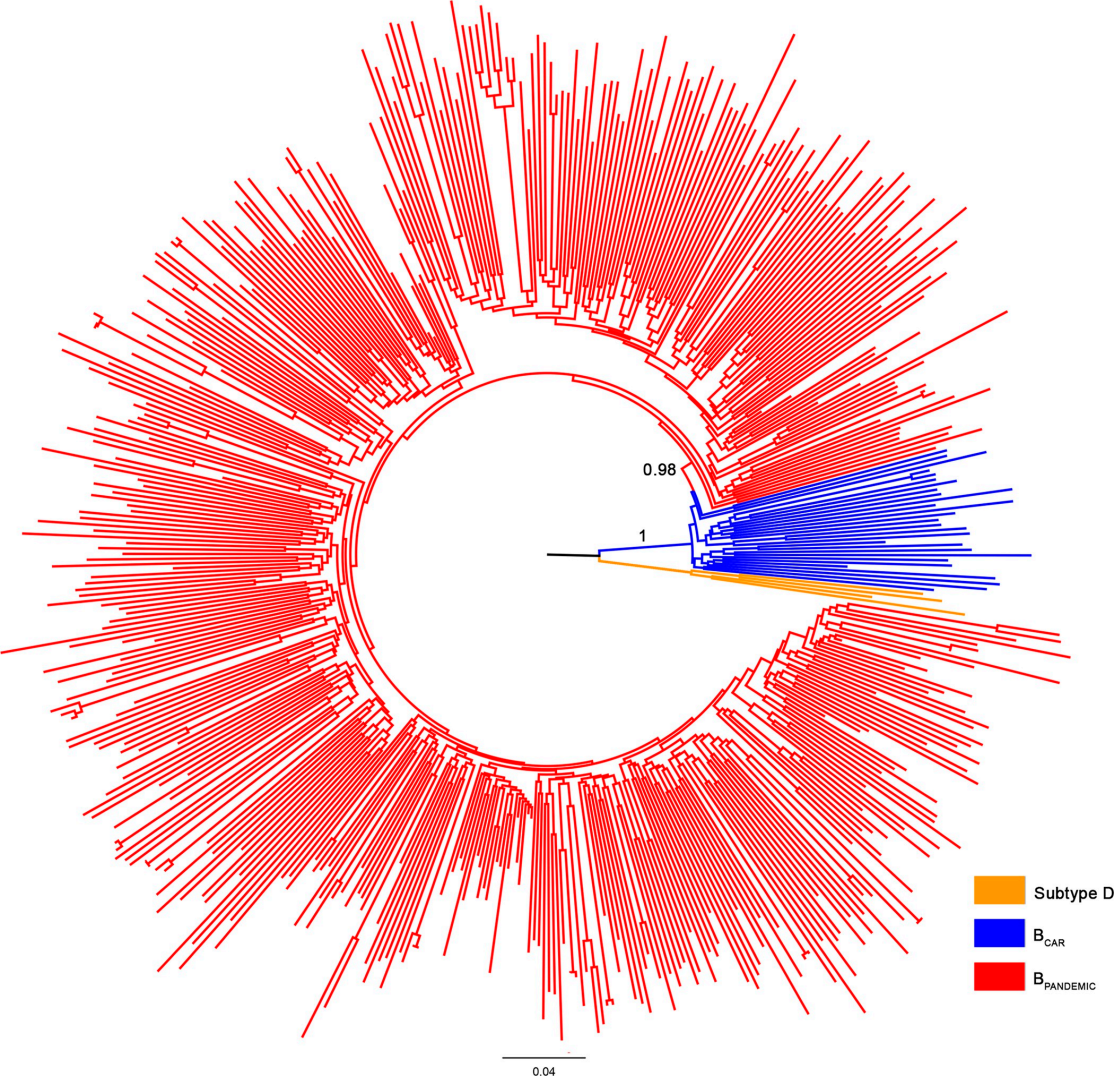
ML phylogenetic tree of 478 HIV-1 subtype B FL genome American sequences. Branches are colored according to their classification in pandemic (B_PANDEMIC_, n = 450) and non-pandemic (B_CAR;_ n = 28) lineages as indicated in the legend at the bottom right. Node support (SH-aLRT) for subtype B and B_PANDEMIC_ monophyletic groups are indicated. The tree was rooted using HIV-1 subtype D sequences. Branch lengths are drawn to scale with the bar at the bottom indicating nucleotide substitutions per site.

From the 506 HIV-1 subtype B FL genome sequences of American origin initially selected, 28 sequences (6%) were identified as putative intra-subtype B recombinants and removed from the final dataset (S1 Table). The ML phylogenetic analysis of the remaining 478 subtype B FL genome American sequences revealed that most Caribbean sequences (82%) branched as basal strains and were classified as B_CAR_ strains, while most sequences from North (97%) and South (99%) America branched in a well-supported (SH-aLRT = 0.98) monophyletic sub-clade and were thus classified as B_PANDEMIC_ strains (Fig 1 and S1 Table). Despite the low number of subtype B FL genome sequences available from the Americas, the estimated relative prevalence of the B_CAR_ lineages in different countries was entirely consistent with previous estimates [6, 8, 9] based on much larger *pol* sequence datasets. Similarly, classification of additional subtype B Caribbean, covering specific regions of *gag* (n = 495), *pol* (n = 775), and *env* (n = 529) genes produced country ratios of B_CAR/_B_PANDEMIC_ sequences akin to their counterparts based on the FL genome (S1 Fig and S1 Table).

### AAs in B_CAR_ and B_PANDEMIC_ modern sequences

In order to identify AAs of different subtype B clades, we compared the FL genome B_PANDEMIC_ sequences (n = 450, sampled between 1978 and 2015*)* of American origin with FL genome (n = 18, sampled between 1983 and 2011), Gag (n = 28), Pol (n = 197), Env (n = 59) and Rev (n = 59, given the superposition of its CDS with Env) B_CAR_ sequences of Caribbean origin (Table 1). Twenty-eight sequences were originally classified as B_CAR_, but 10 were removed for subsequent analyses because were sampled outside the Caribbean region. The same reasoning was used in the expanded dataset, where only B_CAR_ sequences of Caribbean origin were considered. The analysis of translated FL genome sequences identified nine positions that displayed compositions significantly distinct between B_CAR_ and B_PANDEMIC_ datasets, covering structural (one in P6, one in PR and one in RT), regulatory (one in Tat and three in Rev) and accessory (one in Vif and one in Vpu) proteins (Table 2). Expanded B_CAR_ datasets comprising partial regions of Gag, Pol, Env, and Rev encompass four out of the nine AAs previously identified. Three positions (one in PR, one in RT, and one in Rev) had their results endorsed by the additional sequences, while statistical significance in the P6 position was lost. Analyses of the expanded B_CAR_ datasets also identified additional AAs not detected in the FL genome dataset (Table 2), four in Gag (three in P24, positions 27, 120 and 148; and one in P7, position 12) and another in the RT (position 211).

**Table 1:** Sequences used for identification of AAs signatures between B_CAR_ and B_PANDEMIC_ sequences.

Country*	B_PANDEMIC_	B_CAR_						
	FL	Gag		Pol		Env/ Rev		Others
		FL	Expanded	FL	Expanded	FL	Expanded	FL
**DO**	-	4	-	4	101	4	-	4
**HT**	-	5	4	5	-	5	6	5
**JM**	4	4	6	4	69	4	-	4
**TT**	-	5	-	5	-	5	35	5
**BR**	106	-	-	-	-	-	-	-
**US**	306	-	-	-	-	-	-	-
**Others**	34	-	-	-	9	-	-	-
**Total**	450	28		197		59		18

*Country codes are in accordance with ISO 3166–1. FL: Full length.

**Table 2:** Amino acid signatures of B_CAR_ and B_PANDEMIC_ datasets.

Location	Gene	*gag*				*pol*			*vif*	*vpu*	*tat*	*rev*		
	Protein	P24	P24	P24	P7	PR	RT	RT	Vif	Vpu	Tat	Rev		
	Position	27	120	148	12	41	207	211	50	24	23	57	67	102
Ancestors	**B**_**CAR**_	V	S	V	I	K	E	R	R	T	T	A	S	V
	**B**_**PANDEMIC**_	V	S	V	T	K	Q	R	R	T	T	A	S	V
Datasets	**D** *n* = 18	I_89_ V_11_	S_78_ N_17_	V_100_	I_56_ T_22_	K_100_	E_88_ K_6_	K_61_ R_39_	K_89_ R_11_	T_94_ S_6_	N_94_ T_6_	E_88_ A_6_	S_67_ P_33_	I_78_ V_16_
	**B**_**CAR**_ *n* = 18	I_61_ V_39_	S_50_ N_39_	V_61_ T_22_	I_39_ T_33_	K_89_ R_11_	E_50_ A_11_	K_50_ R_50_	K_56_ R_44_	T_83_ S_6_	N_44_ T_44_	A_55_ E_39_	S_83_ P_17_	V_100_
	**B**_**CAR expanded**_	I_71_ V_29_	S_50_ N_25_	V_68_ T_18_	I_54_ T_25_	K_74_ R_23_	E_48_ A_25_	K_54_ R_25_	-	-	-	A_53_ E_32_	-	-
		*n* = 28				*n* = 197						*n* = 59		
	**B**_**PANDEMIC**_ *n* = 450	V_69_ I_31_	N_55_ S_26_	T_66_ V_27_	T_40_ I_28_	R_75_ K_24_	Q_82_ E_9_	R_46_ K_44_	R_76_ K_24_	S_51_ T_45_	T_76_ N_22_	G_40_ E_27_	P_59_ S_39_	I_69_ V_28_
*p*-values adj.	**D/B**_**CAR**_	-	-	-	-	-	-	0.7563	-	-	**0.00391**	**0.0039**	-	**0.0001**
	**D/B**_**PANDEMIC**_	**0.0172**	**0.0461**	**0.0009**	0.3137	**0.0073**	**0.0002**	0.7962	**0.0004**	0.0311	**0.00229**	0.0508	0.1355	-
	**B**_**CAR**_**/B**_**PANDEMIC**_	0.1084	0.3094	0.1084	0.0502	**0.0200**	**0.0008**	0.9670	**0.0255**	**0.0015**	**0.0465**	**0.0300**	**0.0423**	**0.0300**
	**B**_**CAR exp**_**/B**_**PANDEMIC**_	**0.0091**	**0.0091**	**0.0376**	**0.0376**	**0.0001**	**0.0001**	**0.0001**	-	-	-	**0.0001**	-	-

The table details positions identified as bearing amino acidic compositions significantly divergent between B_CAR_ and B_PANDEMIC_ strains. For each position are supplied (1) the residues present in the reconstructed B_CAR_ and B_PANDEMIC_ ancestors; (2) the two most common amino acid observed in that position in Subtype D, B_CAR_, B_CAR Expanded_, and B_PANDEMIC_ datasets accompanied by a number representing its frequency in each group; (3) the adjusted p-values. The sampling range of each sub dataset is indicated in the table. Ancestor sequences were reconstructed employing FL genomes of B_CAR_ (*n* = 18) and B_PANDEMIC_ (*n* = 69) lineages. G (Glycine), P (Proline), A (Alanine), V (Valine), L (Leucine), I (Isoleucine), M (Methionine), C (Cysteine), F (Phenylalanine), Y (Tyrosine), W (Tryptophan), H (Histidine), K (Lysine), R (Arginine), Q (Glutamine), N (Asparagine), E (Glutamic Acid), D (Aspartic Acid), S (Serine), T (Threonine). *p*-values < 0.05 are in bold.

By combining FL and partial genome B_CAR_ datasets, 13 positions were considered as signature positions differentiating B_CAR_ e B_PANDEMIC_ clades: four located on Gag (three in P24, and one in P7); three on Pol (one in the PR and two in the RT); three in Rev; while Vif, Vpu, and Tat each contributed with one position (Table 2). No signature position was identified in Vpr, Env, or Nef. None of the 13 AAs that distinguished contemporary B_CAR_ and B_PANDEMIC_ sequences were found to be invariant sites, with the exception of position Rev 102 in the B_CAR_ lineages. Furthermore, we also observed that for most AA positions, the most common amino acid found in a given subtype B clade corresponds to the second most frequent amino acid in the other clade (Table 2). The exceptions were position 207 in RT that displayed E_48_/A_25_ as the most frequent amino acids in B_CAR_ strains and Q_82_/E_9_ in B_PANDEMIC_ ones, and position 57 in Rev that displayed A_53_/E_32_ as the most frequent amino acids in B_CAR_ strains and G_40_/E_27_ in B_PANDEMIC_ ones.

### AAs in B_CAR_ and B_PANDEMIC_ MRCA sequences

When the reconstructed MRCA sequences of B_CAR_ and B_PANDEMIC_ clades were compared, 21 amino acidic positions differentiated both ancestors (Table 3). Eight of them were located in Gag (one in P17, three in P24, two in P7, and two in P6); four in Pol (all of them in the RT); two in Vif, and four in Env (one in GP120, and three in GP41); while Tat, Rev, and Nef had one position each. Vpr and Vpu presented no difference in the comparisons. Of the 21 amino acidic positions that differentiated both ancestors, only four positions (three in Gag and one in Pol) displayed distinct majoritarian amino acids in the contemporary B_CAR_ and B_PANDEMIC_ datasets and only two of them (positions 12 in P7 and 207 in the RT) correspond to AAs between contemporary B_CAR_ and B_PANDEMIC_ sequences (Table 2). Thus, of the 21 amino acidic positions that differentiated the B_CAR_ and B_PANDEMIC_ ancestors only two continue to distinguish the contemporary descendant sequences. Furthermore, this analysis suggests that most (11/13) AAs that distinguished contemporary B_CAR_ and B_PANDEMIC_ sequences were probably not inherited from their ancestors. It is interesting to note, however, that the most common amino acid in 11 (including the two inherited from ancestors) out of 13 positions associated with the AAs was the same in B_CAR_ and subtype D sequences; while subtype D and B_PANDEMIC_ sequences coincide in only one position.

**Table 3:** Amino acid signatures of HIV-1 B_CAR_ and 210 B_PANDEMIC_ ancestors.

Location	Gene	*gag*								*Pol*				*vif*		*tat*	*rev*	*env*				*nef*
	Protein	P17	P24	P24	P24	P7	P7	P6	P6	RT	RT	RT	RT	Vif	Vif	Tat	Rev	GP120	GP41	GP41	GP41	Nef
	Position	84	15	91	180	12	26	30	42	35	49	122	207	47	66	74	56	363	133	182	209	10
Ancestors	**B**_**CAR**_	T	L	I	D	I	K	Q	R	A	R	K	E	P	V	S	G	P	N	V	L	M
	**B**_**PANDEMIC**_	V	I	V	E	T	R	P	K	V	K	E	Q	T	I	A	S	Q	T	I	R	V
Datasets	**D**	T_83_* V_17_	L_50_ I_50_	V_50_ I_28_	D_61_ E_39_	I_56_ T_22_	K_72_ R_28_	Q_100_	K_100_	T_100_	R_72_ K_28_	E_61_ K_39_	E_89_ K_6_	P_66_ H_28_	V_89_ I_11_	S_100_	G_89_ A_11_	P_83_ S_11_	N_44_ S_44_	I_100_	L_100_	I_89_ L_11_
	**B**_**CAR**_	T_61_ V_39_	L_50_ I_50_	I_72_ V_22_	D_56_ E_44_	I_39_ T_33_	K_61_ R_39_	P_50_ Q_28_	K_72_ R_28_	V_83_ T_11_	K_89_ R_6_	K_67_ P_11_	E_50_ A_11_	T_44_ P_22_	I_67_ V_33_	T_44_ A_39_	S_78_ G_17_	Q_56_ P_17_	T_28_ N_22_	I_56_ V_44_	H_44_ R_39_	L_22_ V_17_
	**B**_**PANDEMIC**_	T_61_ V_39_	I_71_ L_28_	I_57_ V_37_	E_68_ D_32_	T_40_ I_28_	R_55_ K_45_	P_61_ Q_12_	R_53_ K_47_	V_79_ I_12_	K_93_ R_7_	K_62_ E_35_	Q_82_ E_9_	T_58_ P_15_	I_64_ V_35_	T_45_ A_41_	S_96_ G_4_	Q_49_ H_16_	T_45_ N_26_	I_58_ V_41_	H_45_ R_42_	V_18_ L_14_

The table summarizes the most common amino acid for positions in which the reconstructed ancestors of Subtype B and B_PANDEMIC_ diverged, accompanied by a number representing its frequency in each group. G (Glycine), P (Proline), A (Alanine), V (Valine), L (Leucine), I (Isoleucine), M (Methionine), C (Cysteine), F (Phenylalanine), Y (Tyrosine), W (Tryptophan), H (Histidine), K (Lysine), R (Arginine), Q (Glutamine), N (Asparagine), E (Glutamic Acid), D (Aspartic Acid), S (Serine), T (Threonine).

### Predicted phenotypic characteristics of B_CAR_ and B_PANDEMIC_ strains

**Fig 2:**
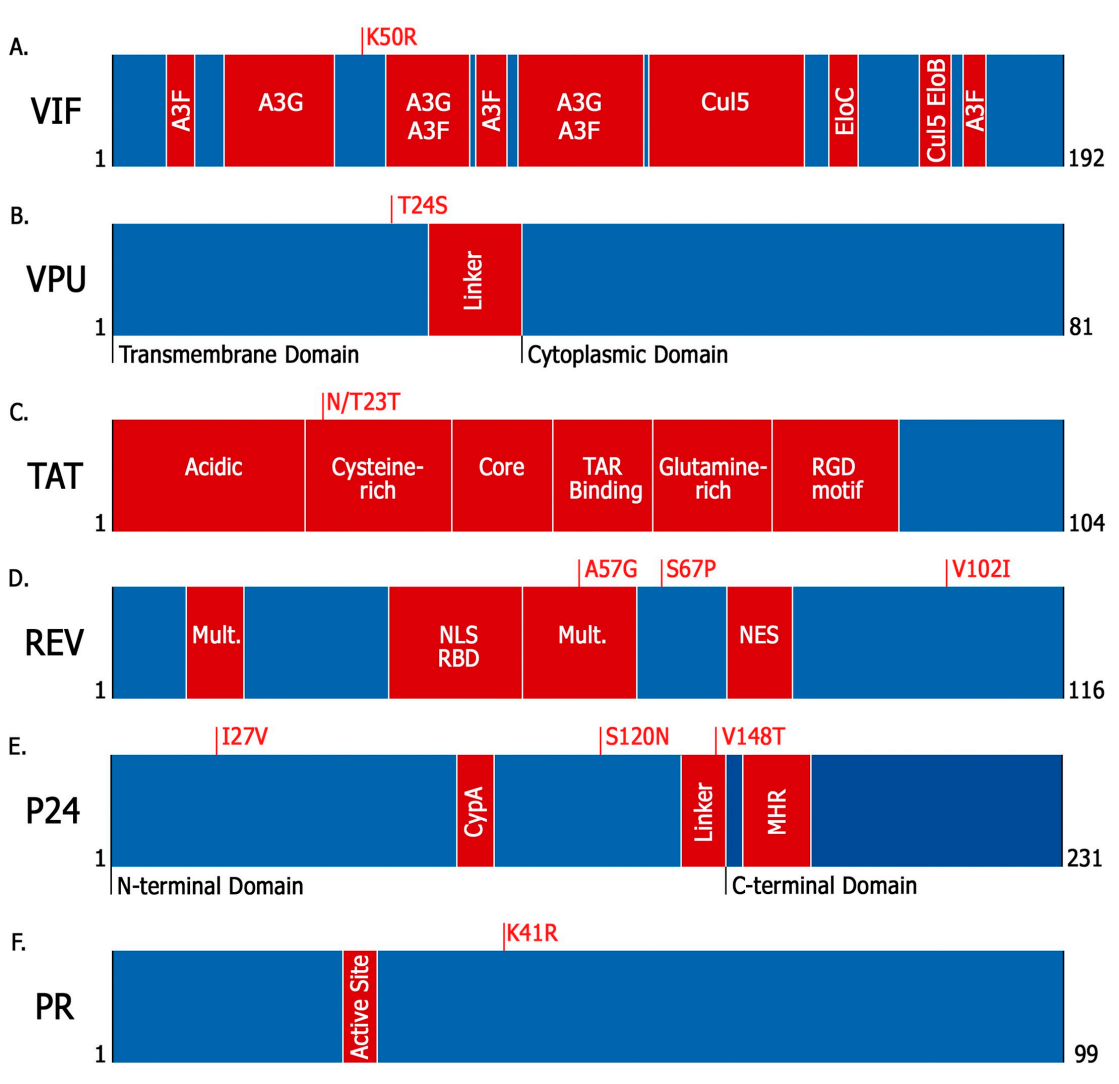
Mapping of the identified AAs between B_CAR_ and B_PANDEMIC_ on the accessory, regulatory and structural HIV-1 proteins. For all proteins, their functional domains are represented; (**A**) Vif: regions responsible for the binding to APOBEC3G (A3G), APOBEC3F (A3F), Cullin 5 (Cul5), Elongin B (EloB), and Elongin C (EloC); (**B**) Vpu: the transmembrane domain, the cytoplasmic domain, and the linker region between them; (**C**) Tat: the N‐terminal acidic domain, the cysteine-rich domain, the hydrophobic core domain, the TAR binding domain, the glutamine-rich domain, and the RGD motif; (**D**) Rev: the RNA binding domain (RBD), that also functions as a nuclear localization signal (NLS), the nuclear exporting signal (NES), and sequences responsible for its multimerization (Mult.); (E) P24: the N-terminal domain and the C-terminal domain, connected by the inter-domain linker, the region mainly responsible for the interaction with cyclophilin A (CypA), and the major homology region (MHR); (F) Protease (PR): its active site.

Most AAs that distinguish the B_CAR_ and B_PANDEMIC_ strains were located outside domains or sites previously reported to be essential for protein function [40, 57–66] (Fig 2). The sole exception was position 23, located in Tat cysteine-rich domain (22–37), and reported as one of the three major sites of p53-derived restriction of Tat mediated by PKR phosphorylation [67]. We also evaluate the frequency of several polymorphisms in Vif, Vpr, Nef and Rev previously associated with long-term non-progressors (LTNPs) HIV-1 infected subjects and differential protein function in vitro and ex vivo [39–50] (Table 4). Analysis of amino acid composition at those positions failed to detect significant differences between B_CAR_ and B_PANDEMIC_ datasets, except for position 77 in Vpr that showed a significantly higher prevalence of the R77Q mutation in B_CAR_ (83%) in comparison with B_PANDEMIC_ (48%) sequences [45]. None of the methods here employed (Geno2Pheno algorithm, the 11/25 rule, or the combination of R at position 25 of V3 and a net charge of ≥ 5) pointed out to significant difference in the frequency of CXCR4 tropic viral variants, typically associated with a more rapid disease progression [68, 69], between the B_PANDEMIC_ and B_CAR_ env datasets (S3 Table). Finally, our analysis of HIV-1 pol sequences from drug naïve subjects from the Caribbean also failed to detect significant differences in the prevalence of SDRMs between B_CAR_ and B_PANDEMIC_ datasets (S4 Table).

**Table 4:** Prevalence of polymorphisms in B_CAR_ and B_PANDEMIC_ Vif, Vpr, Nef and Rev sequences associated with slow HIV-1 disease progression and differential function.

Polymorphism	B_CAR_		B_PANDEMIC_		*p-*value
	# seq. Analyzed	# seq. with polymorphism (%)	# seq. analyzed	# seq. with polymorphism (%)	
**RevE74P**	18	0	450	36 (8%)	*-*
**RevL78I**	18	0	450	43 (10%)	*-*
**RevV104L**	18	0	450	31 (7%)	*-*
**RevS106P**	18	1 (6%)	450	28 (6%)	0.908
**VifI107T**	18	0	450	0	-
**VifR132S**	18	0	450	135 (30%)	-
**VprQ3R**	18	3 (17%)	450	42 (9%)	0.300
**VprQ44R**	18	0	450	0	-
**VprF72L**	18	0	450	0	-
**VprR77Q**	18	15 (83%)	450	218 (48%)	**0.003**
**VprI83V**	18	0	450	8 (2%)	-
**NefR22Q**	18	3	450	49 (11%)	
**NefL58V**	18	3	450	45 (10%)	
**NefK94E**	18	0	450	11 (2%)	-
**NefH116N**	18	8 (44%)	450	123 (27%)	0.112

*p-*values < 0.05 are in bold.

## Discussion

The current work suggests that the hypothesis that viral genetic determinants shaped the remarkable differences in the geographic dissemination pattern of the HIV-1 B_PANDEMIC_ and B_CAR_ strains is highly unlikely. Among over 3,000 positions analyzed across nine genes coded by the HIV-1 genome, we detected only 13 AAs distinguishing the B_CAR_ and B_PANDEMIC_ clades. All AAs that did differentiate the B_CAR_ and B_PANDEMIC_ clades correspond to sites with distinct degrees of polymorphism and not to invariant (or highly conserved) sites. Furthermore, for 11 out of 13 AAs positions, the most common amino acid found in a given subtype B variant corresponds to the second most frequent amino acid in the other variant. Our study also suggests that 11 out of 13 AAs that distinguished contemporary B_CAR_ and B_PANDEMIC_ sequences were probably not inherited from their ancestors and that most (19/21) amino acid differences inferred between the B_CAR_ and B_PANDEMIC_ ancestors evolved into polymorphic sites with quite comparable compositions in modern descendant sequences. Finally, nearly all AAs identified were located outside functionally relevant protein domains.

Our data supports that B_CAR_ and B_PANDEMIC_ strains are probably not distinguished by functionally relevant AAs in structural genes. Analyzes of both FL and expanded partial *env* sequences failed to detect AAs in this variable genomic region. Furthermore, no significant differences in the frequency of CCR5/CXCR4 tropic variants were detected between B_CAR_ and B_PANDEMIC_ sequences, supporting not great variation in the chemokine receptor usage between pandemic and non-pandemic subtype B strains. These results are fully consistent with a previous study that demonstrate that the *env* V3 consensus sequence of B_CAR_ strains from Trinidad and Tobago differs by few amino acids from the B_PANDEMIC_ V3 consensus and that no phenotypic features, including syncytium induction, neutralization profiles, and chemokine receptor usage, distinguish both subtype B lineages [19]. Furthermore, all AAs in Gag and Pol that distinguishing B_CAR_ and B_PANDEMIC_ sequences were located outside known conserved protein functional domains.

By contrasting, a few interesting differences between B_CAR_ and B_PANDEMIC_ strains were observed in non-structural genes. The single AAs in position 23 of Tat is located in a cysteine-rich domain and has been reported as one of the three major sites of p53-derived restriction of Tat mediated by PKR phosphorylation [67]. The presence of an N residue in that position, that is the most prevalent amino acid in subtypes A, C, D and B_CAR_ (44%) strains, but not in B_PANDEMIC_ ones (22%), have been associated with increased Tat transactivation, probably through enhanced P-TEFb binding [67, 70]. We also detect much higher frequency of the naturally occurring variation Vpr R77Q in B_CAR_ (83%) respect to B_PANDEMIC_ (48%) sequences. That mutation, that also predominates in subtypes A, C, D, G, and H, reduces apoptosis and CD4 T-cell depletion in *ex vivo*-infected cells and is much more prevalent in subtype B-infected LTNPs individuals (75–90%) than in subjects with progressive HIV disease (33–42%) [45]. The similar genetic composition of B_CAR_ and several pandemic HIV-1 clades (subtypes A, C and D) at positions Tat23 and Vpr77 argued against the hypothesis that differences at such positions resulted in a more restricted dispersion of B_CAR_ compared with the B_PANDEMIC_ strains.

Despite the very small size (n = 18) of the B_CAR_ FL genome dataset here used, some evidences support that the B_CAR_ genetic variability was not severely underestimated in this dataset and that the B_CAR_ consensus sequence obtained was probably not biased by the low number of FL genomic sequences available. First, the most common amino acid recovered in most positions from extended datasets was fully coincident with the one detected in the FL dataset. Expanded and FL datasets converged in 99.7% (297/298) of Gag amino acid positions, 99.1% (336/339) of Pol positions, and 97.8% (683/698) of Env positions analyzed. Second, the degree of polymorphism of the 13 AAs positions that distinguished B_CAR_ and B_PANDEMIC_ sequences was roughly comparable in FL and expanded B_CAR_ datasets. The paucity of FL B_CAR_ strains, however, might have restricted our ability to detect some additional AAs between B_CAR_ and B_PANDEMIC_ sequences. By increasing the number of B_CAR_ sequences we failed to recover new AAs between B_CAR_ and B_PANDEMIC_ *env* sequences, but we detected four additional AAs in Gag and one in Pol, increasing the overall number of AAs from three to eight in those genomic regions.

The low number of FL B_CAR_ sequences used might have also introduced some bias on the reconstruction of the MRCA sequences. According to our analysis, of the 13 AAs detected in modern B_CAR_ and B_PANDEMIC_ sequences, only two matched with divergent sites between the B_CAR_ and B_PANDEMIC_ ancestors. This observation support that most genetic differences between the B_CAR_ and B_PANDEMIC_ ancestors evolved toward positions with similar amino acid composition in modern subtype B sequences and that most AAs in modern B_CAR_ and B_PANDEMIC_ sequences arose during subsequent evolution and diversification of subtype B lineages. An inspection of the amino acid composition at those 13 positions in the related subtype D clade, however, supports a different scenario. We observed that B_CAR_ and subtype D sequences displayed the same prevalent amino acid in most (11/13) AAs positions, consistent with genetic identity inherited from the common B/D ancestor. In sharp contrast, the B_PANDEMIC_ and subtype D sequences displayed the same prevalent amino acid in only one AAs position. Therefore, our reconstruction of the MRCA sequences may have underestimated the number of B_CAR_/B_PANDEMIC_ AAs inherited from the ancestors.

In summary, albeit some mutations fixed in the HIV-1 B_PANDEMIC_ ancestral strain could potentially have some phenotypic impact on viral transmissibility, the absence of stringent AAs distinguishing modern B_CAR_ and B_PANDEMIC_ variants and the similar amino acid composition between B_CAR_ and other group M subtype pandemic variants at key sites indicates that viral genetic determinants were probably not the main factor shaping the divergent pattern of geographic spread of B_CAR_ and B_PANDEMIC_ variants. The successful dissemination of B_CAR_ strains in some Caribbean countries that exhibit the highest HIV-1 prevalence rates outside of Africa [6, 11] also argues against the hypothesis of a reduced B_CAR_ viral fitness. These results support that stochastic events leading to the introduction of B_PANDEMIC_ ancestor into globally connected populations were the most probable driving force behind its pandemic dissemination and substantiate the crucial need for continued molecular surveillance of HIV-1 transmission on key populations worldwide.

## Supporting information

S1 Fig ML phylogenetic trees of HIV-1 subtype B American sequences on specific regions of *gag*, *pol*, and *env*. Partial HIV-1 sequences covering (A) *gag* (1,264 to 2,148), (B) *env* (6,450 to 8,480), (C) *pol* (2,253 to 3,272), and (D) *pol* from drug naïve individuals (2,253 to 3,272) were classified into B_PANDEMIC_ (red branches) and B_CAR_ (blue branches) lineages according to the topology obtained in each tree. Node support (SH-aLRT) for subtype B and B_PANDEMIC_ monophyletic groups are indicated. The trees were rooted using HIV-1 subtype D sequences. Branch lengths are drawn to scale with the bar at the bottom indicating nucleotide substitutions per site.
(PDF)

S1 Table Classification of the HIV-1 subtype B sequences in the B_PANDEMIC_ or B_CAR_ clades. The table summarizes the results of the classification of the full-length (FL) and partial HIV-1 subtype B sequences in the B_PANDEMIC_ or B_CAR_ based on their placement on ML phylogenetic trees displayed in Fig 1 and S1 Fig. All sub-datasets are accompanied by their sampling range. *Country codes are in accordance with ISO 3166–1.
(PDF)

S2 Table HIV-1 subtype D full-length genome sequences. The table summarize the full-length (FL) HIV-1 Subtype D sequences used in our study and their sampling range. * Country codes are in accordance with ISO 3166–1.
(PDF)

S3 Table Predicted co-receptor usage by B_CAR_ and B_PANDEMIC_ *env* sequences. The table summarizes the predicted usage of chemokines receptors CCR5 and CXCR4 based on different criteria: 1) the Geno2Pheno algorithm, which classifies the sequences between R5 variants or X4 and R5X4 dual-tropic variants; 2) the 11/25 Rule, which asses the presence of arginine (R) or lysine (K) at position 11 of *env* V3 sequences and/or K at position 25; 3) the combination of R at position 25 of V3 and a net charge of ≥ 5.
(PDF)

S4 Table Prevalence of transmitted drug-resistance mutations in B_CAR_ and B_PANDEMIC_ PR/RT sequences. The table summarizes the surveillance drug-resistance mutations (SDRM) identified in PR/RT B_CAR_ and B_PANDEMIC_ sequences retrieved from drug naïve subjects. PI (protease inhibitor), NRTI (nucleoside analog reverse-transcriptase inhibitors), NNRTI (non-nucleoside analog reverse-transcriptase inhibitor). The *p*-values obtained in chi-squared tests are listed in the last column.
(PDF)

S1 File (PDF)

## References

[1] Tebit DM, Arts EJ. Tracking a century of global expansion and evolution of HIV to drive understanding and to combat disease. Lancet Infect Dis. 2011;11(1):45–56. 21126914 10.1016/S1473-3099(10)70186-9

[2] Hemelaar J, Elangovan R, Yun J, Dickson-Tetteh L, Fleminger I, Kirtley S, et al Global and regional molecular epidemiology of HIV-1, 1990–2015: a systematic review, global survey, and trend analysis. Lancet Infect Dis. 2019;19(2):143–55. 10.1016/S1473-3099(18)30647-9 30509777

[3] Faria NR, Rambaut A, Suchard MA, Baele G, Bedford T, Ward MJ, et al HIV epidemiology. The early spread and epidemic ignition of HIV-1 in human populations. Science. 2014;346(6205):56–61. 10.1126/science.1256739 25278604 PMC4254776

[4] Gilbert MT, Rambaut A, Wlasiuk G, Spira TJ, Pitchenik AE, Worobey M. The emergence of HIV/AIDS in the Americas and beyond. Proc Natl Acad Sci U S A. 2007;104(47):18566–70. 10.1073/pnas.0705329104 17978186 PMC2141817

[5] Junqueira DM, de Medeiros RM, Matte MC, Araújo LA, Chies JA, Ashton-Prolla P, et al Reviewing the history of HIV-1: spread of subtype B in the Americas. PLoS One. 2011;6(11):e27489 10.1371/journal.pone.0027489 22132104 PMC3223166

[6] Cabello M, Mendoza Y, Bello G. Spatiotemporal dynamics of dissemination of non-pandemic HIV-1 subtype B clades in the Caribbean region. PLoS One. 2014;9(8):e106045 10.1371/journal.pone.0106045 25148215 PMC4141835

[7] Bello G, Arantes I, Lacoste V, Ouka M, Boncy J, Césaire R, et al Phylogeographic Analyses Reveal the Early Expansion and Frequent Bidirectional Cross-Border Transmissions of Non-pandemic HIV-1 Subtype B Strains in Hispaniola. Front Microbiol. 2019;10:1340 10.3389/fmicb.2019.01340 31333594 PMC6622406

[8] Cabello M, Junqueira DM, Bello G. Dissemination of nonpandemic Caribbean HIV-1 subtype B clades in Latin America. AIDS. 2015;29(4):483–92. 10.1097/QAD.0000000000000552 25630042

[9] Cabello M, Romero H, Bello G. Multiple introductions and onward transmission of non-pandemic HIV-1 subtype B strains in North America and Europe. Sci Rep. 2016;6:33971 10.1038/srep33971 27653834 PMC5032033

[10] Divino F, de Lima Guerra Corado A, Gomes Naveca F, Stefani MM, Bello G. High Prevalence and Onward Transmission of Non-Pandemic HIV-1 Subtype B Clades in Northern and Northeastern Brazilian Regions. PLoS One. 2016;11(9):e0162112 10.1371/journal.pone.0162112 27603317 PMC5014447

[11] Bello G, Nacher M, Divino F, Darcissac E, Mir D, Lacoste V. The HIV-1 Subtype B Epidemic in French Guiana and Suriname Is Driven by Ongoing Transmissions of Pandemic and Non-pandemic Lineages. Front Microbiol. 2018;9:1738 10.3389/fmicb.2018.01738 30108576 PMC6079251

[12] Jaffe HW, Darrow WW, Echenberg DF, O'Malley PM, Getchell JP, Kalyanaraman VS, et al The acquired immunodeficiency syndrome in a cohort of homosexual men. A six-year follow-up study. Ann Intern Med. 1985;103(2):210–4. 10.7326/0003-4819-103-2-210 2990275

[13] Stevens CE, Taylor PE, Zang EA, Morrison JM, Harley EJ, Rodriguez de Cordoba S, et al Human T-cell lymphotropic virus type III infection in a cohort of homosexual men in New York City. JAMA. 1986;255(16):2167–72. 3007789

[14] Quinn TC, Wawer MJ, Sewankambo N, Serwadda D, Li C, Wabwire-Mangen F, et al Viral load and heterosexual transmission of human immunodeficiency virus type 1. Rakai Project Study Group. N Engl J Med. 2000;342(13):921–9. 10.1056/NEJM200003303421303 10738050

[15] Alizon S, von Wyl V, Stadler T, Kouyos RD, Yerly S, Hirschel B, et al Phylogenetic approach reveals that virus genotype largely determines HIV set-point viral load. PLoS Pathog. 2010;6(9):e1001123 10.1371/journal.ppat.1001123 20941398 PMC2947993

[16] Blanquart F, Wymant C, Cornelissen M, Gall A, Bakker M, Bezemer D, et al Viral genetic variation accounts for a third of variability in HIV-1 set-point viral load in Europe. PLoS Biol. 2017;15(6):e2001855 10.1371/journal.pbio.2001855 28604782 PMC5467800

[17] Bertels F, Marzel A, Leventhal G, Mitov V, Fellay J, Günthard HF, et al Dissecting HIV Virulence: Heritability of Setpoint Viral Load, CD4+ T-Cell Decline, and Per-Parasite Pathogenicity. Mol Biol Evol. 2018;35(1):27–37. 10.1093/molbev/msx246 29029206 PMC5850767

[18] Mitov V, Stadler T. A Practical Guide to Estimating the Heritability of Pathogen Traits. Mol Biol Evol. 2018. 10.1093/molbev/msx328 PMC5850476 29329426

[19] Cleghorn FR, Jack N, Carr JK, Edwards J, Mahabir B, Sill A, et al A distinctive clade B HIV type 1 is heterosexually transmitted in Trinidad and Tobago. Proc Natl Acad Sci U S A. 2000;97(19):10532–7. 10.1073/pnas.97.19.10532 10984542 PMC27059

[20] Collins-Fairclough AM, Charurat M, Nadai Y, Pando M, Avila MM, Blattner WA, et al Significantly longer envelope V2 loops are characteristic of heterosexually transmitted subtype B HIV-1 in Trinidad. PLoS One. 2011;6(6):e19995. 21698149 10.1371/journal.pone.0019995 PMC3117786

[21] Gaschen B, Kuiken C, Korber B, Foley B. Retrieval and on-the-fly alignment of sequence fragments from the HIV database. Bioinformatics. 2001;17(5):415–8. 10.1093/bioinformatics/17.5.415 11331235

[22] Martin DP, Murrell B, Golden M, Khoosal A, Muhire B. RDP4: Detection and analysis of recombination patterns in virus genomes. Virus Evol. 2015;1(1):vev003 10.1093/ve/vev003 27774277 PMC5014473

[23] Guindon S, Dufayard JF, Lefort V, Anisimova M, Hordijk W, Gascuel O. New algorithms and methods to estimate maximum-likelihood phylogenies: assessing the performance of PhyML 3.0. Syst Biol. 2010;59(3):307–21. 10.1093/sysbio/syq010 20525638

[24] Guindon S, Lethiec F, Duroux P, Gascuel O. PHYML Online—a web server for fast maximum likelihood-based phylogenetic inference. Nucleic Acids Res. 2005;33(Web Server issue):W557–9. 10.1093/nar/gki352 15980534 PMC1160113

[25] Anisimova M, Gascuel O. Approximate likelihood-ratio test for branches: A fast, accurate, and powerful alternative. Syst Biol. 2006;55(4):539–52. 10.1080/10635150600755453 16785212

[26] A R. FigTree v1.4: Tree Figure Drawing Tool. Available from: http://treebioedacuk/software/figtree/. 2009.

[27] Fu L, Niu B, Zhu Z, Wu S, Li W. CD-HIT: accelerated for clustering the next-generation sequencing data. Bioinformatics. 2012;28(23):3150–2. 10.1093/bioinformatics/bts565 23060610 PMC3516142

[28] Huang Y, Niu B, Gao Y, Fu L, Li W. CD-HIT Suite: a web server for clustering and comparing biological sequences. Bioinformatics. 2010;26(5):680–2. 10.1093/bioinformatics/btq003 20053844 PMC2828112

[29] Drummond AJ, Nicholls GK, Rodrigo AG, Solomon W. Estimating mutation parameters, population history and genealogy simultaneously from temporally spaced sequence data. Genetics. 2002;161(3):1307–20. 12136032 10.1093/genetics/161.3.1307 PMC1462188

[30] Drummond AJ, Rambaut A. BEAST: Bayesian evolutionary analysis by sampling trees. BMC Evol Biol. 2007;7:214 10.1186/1471-2148-7-214 17996036 PMC2247476

[31] Suchard MA, Rambaut A. Many-core algorithms for statistical phylogenetics. Bioinformatics. 2009;25(11):1370–6. 10.1093/bioinformatics/btp244 19369496 PMC2682525

[32] Rodríguez F, Oliver JL, Marín A, Medina JR. The general stochastic model of nucleotide substitution. J Theor Biol. 1990;142(4):485–501. 10.1016/s0022-5193(05)80104-3 2338834

[33] Drummond AJ, Ho SY, Phillips MJ, Rambaut A. Relaxed phylogenetics and dating with confidence. PLoS Biol. 2006;4(5):e88 10.1371/journal.pbio.0040088 16683862 PMC1395354

[34] Drummond AJ, Rambaut A, Shapiro B, Pybus OG. Bayesian coalescent inference of past population dynamics from molecular sequences. Mol Biol Evol. 2005;22(5):1185–92. 10.1093/molbev/msi103 15703244

[35] A R, MA S, D X, AJ D. Tracer v1.6, Available from http://tree.bio.ed.ac.uk/software/tracer/ 2014.

[36] Gouy M, Guindon S, Gascuel O. SeaView version 4: A multiplatform graphical user interface for sequence alignment and phylogenetic tree building. Mol Biol Evol. 2010;27(2):221–4. 19854763 10.1093/molbev/msp259

[37] Korber B, Myers G. Signature pattern analysis: a method for assessing viral sequence relatedness. AIDS Res Hum Retroviruses. 1992;8(9):1549–60. 1457200 10.1089/aid.1992.8.1549

[38] Team RC. R: A Language and Environment for Statistical Computing. R Foundation for Statistical Computing; 2018.

[39] Iversen AK, Shpaer EG, Rodrigo AG, Hirsch MS, Walker BD, Sheppard HW, et al Persistence of attenuated rev genes in a human immunodeficiency virus type 1-infected asymptomatic individual. J Virol. 1995;69(9):5743–53. 10.1128/JVI.69.9.5743-5753.1995 7637019 PMC189435

[40] Churchill MJ, Chiavaroli L, Wesselingh SL, Gorry PR. Persistence of attenuated HIV-1 rev alleles in an epidemiologically linked cohort of long-term survivors infected with nef-deleted virus. Retrovirology. 2007;4:43 10.1186/1742-4690-4-43 17601342 PMC1933581

[41] Hassaïne G, Agostini I, Candotti D, Bessou G, Caballero M, Agut H, et al Characterization of human immunodeficiency virus type 1 vif gene in long-term asymptomatic individuals. Virology. 2000;276(1):169–80. 10.1006/viro.2000.0543 11022005

[42] Peng J, Ao Z, Matthews C, Wang X, Ramdahin S, Chen X, et al A naturally occurring Vif mutant (I107T) attenuates anti-APOBEC3G activity and HIV-1 replication. J Mol Biol. 2013;425(16):2840–52. 10.1016/j.jmb.2013.05.015 23707381

[43] Somasundaran M, Sharkey M, Brichacek B, Luzuriaga K, Emerman M, Sullivan JL, et al Evidence for a cytopathogenicity determinant in HIV-1 Vpr. Proc Natl Acad Sci U S A. 2002;99(14):9503–8. 10.1073/pnas.142313699 12093916 PMC123170

[44] Zhao Y, Chen M, Wang B, Yang J, Elder RT, Song XQ, et al Functional conservation of HIV-1 Vpr and variability in a mother-child pair of long-term non-progressors. Virus Res. 2002;89(1):103–21. 10.1016/s0168-1702(02)00127-2 12367754

[45] Lum JJ, Cohen OJ, Nie Z, Weaver JG, Gomez TS, Yao XJ, et al Vpr R77Q is associated with long-term nonprogressive HIV infection and impaired induction of apoptosis. J Clin Invest. 2003;111(10):1547–54. 10.1172/JCI16233 12750404 PMC155040

[46] Mologni D, Citterio P, Menzaghi B, Zanone Poma B, Riva C, Broggini V, et al Vpr and HIV-1 disease progression: R77Q mutation is associated with long-term control of HIV-1 infection in different groups of patients. AIDS. 2006;20(4):567–74. 10.1097/01.aids.0000210611.60459.0e 16470121

[47] Caly L, Saksena NK, Piller SC, Jans DA. Impaired nuclear import and viral incorporation of Vpr derived from a HIV long-term non-progressor. Retrovirology. 2008;5:67 10.1186/1742-4690-5-67 18638397 PMC2515335

[48] Jin SW, Alsahafi N, Kuang XT, Swann SA, Toyoda M, Göttlinger H, et al Natural HIV-1 Nef Polymorphisms Impair SERINC5 Downregulation Activity. Cell Rep. 2019;29(6):1449–57.e5. 31693887 10.1016/j.celrep.2019.10.007 PMC6925589

[49] Corró G, Rocco CA, De Candia C, Catano G, Turk G, Mangano A, et al Genetic and functional analysis of HIV type 1 nef gene derived from long-term nonprogressor children: association of attenuated variants with slow progression to pediatric AIDS. AIDS Res Hum Retroviruses. 2012;28(12):1617–26. 22583022 10.1089/AID.2012.0020

[50] Premkumar DR, Ma XZ, Maitra RK, Chakrabarti BK, Salkowitz J, Yen-Lieberman B, et al The nef gene from a long-term HIV type 1 nonprogressor. AIDS Res Hum Retroviruses. 1996;12(4):337–45. 8906995 10.1089/aid.1996.12.337

[51] Lengauer T, Sander O, Sierra S, Thielen A, Kaiser R. Bioinformatics prediction of HIV coreceptor usage. Nat Biotechnol. 2007;25(12):1407–10. 18066037 10.1038/nbt1371

[52] De Jong JJ, De Ronde A, Keulen W, Tersmette M, Goudsmit J. Minimal requirements for the human immunodeficiency virus type 1 V3 domain to support the syncytium-inducing phenotype: analysis by single amino acid substitution. J Virol. 1992;66(11):6777–80. 1404617 10.1128/jvi.66.11.6777-6780.1992 PMC240176

[53] Fouchier RA, Brouwer M, Broersen SM, Schuitemaker H. Simple determination of human immunodeficiency virus type 1 syncytium-inducing V3 genotype by PCR. J Clin Microbiol. 1995;33(4):906–11. 10.1128/JCM.33.4.906-911.1995 7790458 PMC228065

[54] Hoffman NG, Seillier-Moiseiwitsch F, Ahn J, Walker JM, Swanstrom R. Variability in the human immunodeficiency virus type 1 gp120 Env protein linked to phenotype-associated changes in the V3 loop. J Virol. 2002;76(8):3852–64. 10.1128/JVI.76.8.3852-3864.2002 11907225 PMC136063

[55] Fouchier RA, Groenink M, Kootstra NA, Tersmette M, Huisman HG, Miedema F, et al Phenotype-associated sequence variation in the third variable domain of the human immunodeficiency virus type 1 gp120 molecule. J Virol. 1992;66(5):3183–7. 10.1128/JVI.66.5.3183-3187.1992 1560543 PMC241084

[56] Rhee SY, Gonzales MJ, Kantor R, Betts BJ, Ravela J, Shafer RW. Human immunodeficiency virus reverse transcriptase and protease sequence database. Nucleic Acids Res. 2003;31(1):298–303. 10.1093/nar/gkg100 12520007 PMC165547

[57] Cruz NV, Amorim R, Oliveira FE, Speranza FA, Costa LJ. Mutations in the nef and vif genes associated with progression to AIDS in elite controller and slow-progressor patients. J Med Virol. 2013;85(4):563–74. 23417613 10.1002/jmv.23512

[58] Kikuchi T, Iwabu Y, Tada T, Kawana-Tachikawa A, Koga M, Hosoya N, et al Anti-APOBEC3G activity of HIV-1 Vif protein is attenuated in elite controllers. J Virol. 2015;89(9):4992–5001. 25717111 10.1128/JVI.03464-14 PMC4403497

[59] Andrew A, Strebel K. HIV-1 Vpu targets cell surface markers CD4 and BST-2 through distinct mechanisms. Mol Aspects Med. 2010;31(5):407–17. 20858517 10.1016/j.mam.2010.08.002 PMC2967615

[60] Le Noury DA, Mosebi S, Papathanasopoulos MA, Hewer R. Functional roles of HIV-1 Vpu and CD74: Details and implications of the Vpu-CD74 interaction. Cell Immunol. 2015;298(1–2):25–32. 26321123 10.1016/j.cellimm.2015.08.005

[61] Vercruysse T, Daelemans D. HIV-1 Rev multimerization: mechanism and insights. Curr HIV Res. 2013;11(8):623–34. 10.2174/1570162x12666140307094603 24606219

[62] Chen JH, Wong KH, Chan KC, To SW, Chen Z, Yam WC. Phylodynamics of HIV-1 subtype B among the men-having-sex-with-men (MSM) population in Hong Kong. PLoS One. 2011;6(9):e25286 10.1371/journal.pone.0025286 21966483 PMC3178636

[63] Das K, Arnold E. HIV-1 reverse transcriptase and antiviral drug resistance. Part 1. Curr Opin Virol. 2013;3(2):111–8. 10.1016/j.coviro.2013.03.012 23602471 PMC4097814

[64] Das K, Arnold E. HIV-1 reverse transcriptase and antiviral drug resistance. Part 2. Curr Opin Virol. 2013;3(2):119–28. 23602470 10.1016/j.coviro.2013.03.014 PMC4097817

[65] Su CT, Koh DW, Gan SK. Reviewing HIV-1 Gag Mutations in Protease Inhibitors Resistance: Insights for Possible Novel Gag Inhibitor Designs. Molecules. 2019;24(18). 10.3390/molecules24183243 PMC6767625 31489889

[66] Voshavar C. Protease Inhibitors for the Treatment of HIV/AIDS: Recent Advances and Future Challenges. Curr Top Med Chem. 2019;19(18):1571–98. 31237209 10.2174/1568026619666190619115243

[67] Yoon CH, Kim SY, Byeon SE, Jeong Y, Lee J, Kim KP, et al p53-derived host restriction of HIV-1 replication by protein kinase R-mediated Tat phosphorylation and inactivation. J Virol. 2015;89(8):4262–80. 25653431 10.1128/JVI.03087-14 PMC4442402

[68] Connor RI, Sheridan KE, Ceradini D, Choe S, Landau NR. Change in coreceptor use correlates with disease progression in HIV-1—infected individuals. J Exp Med. 1997;185(4):621–8. 9034141 10.1084/jem.185.4.621 PMC2196142

[69] Regoes RR, Bonhoeffer S. The HIV coreceptor switch: a population dynamical perspective. Trends Microbiol. 2005;13(6):269–77. 10.1016/j.tim.2005.04.005 15936659

[70] Reza SM, Shen LM, Mukhopadhyay R, Rosetti M, Pe'ery T, Mathews MB. A naturally occurring substitution in human immunodeficiency virus Tat increases expression of the viral genome. J Virol. 2003;77(15):8602–6. 10.1128/jvi.77.15.8602-8606.2003 12857933 PMC165250

