# Supplementary material for: Few amino acid signatures distinguish HIV-1 subtype B pandemic and non-pandemic strains
Source: PLoS One. 2020 Sep 22;15(9):e0238995. doi: 10.1371/journal.pone.0238995 (PMC7508567; doi:10.1371/journal.pone.0238995)
Supplement: S1 Fig — Partial HIV-1 sequences covering (A) gag (1,264 to 2,148), (B) env (6,450 to 8,480), (C) pol (2,253 to 3,272), and (D) pol from drug naïve individuals (2,253 to 3,272) were classified into BPANDEMIC (red branches) and BCAR (blue branches) lineages according to the topology obtained in each tree. Node support (SH-aLRT) for subtype B and BPANDEMIC monophyletic groups are indicated. The trees were rooted using HIV-1 subtype D sequences. Branch lengths are drawn to scale with the bar at the bottom indicating nucleotide substitutions per site. (PDF) [file pone.0238995.s001.pdf]

Supplementary Figures:

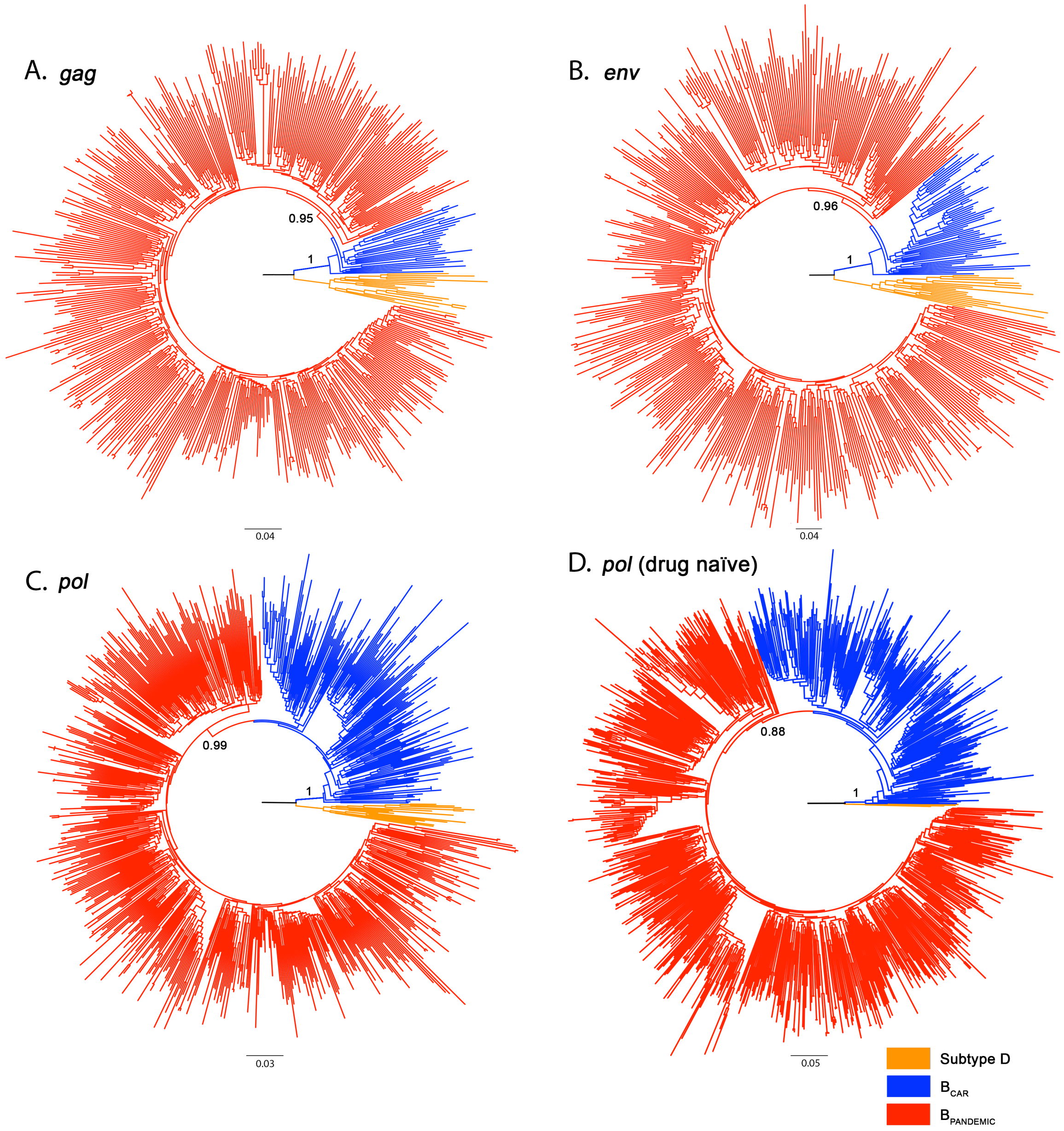

Figure S1: ML phylogenetic trees of HIV-1 subtype B American sequences on specific regions of *gag*, *pol*, and *env*. Partial HIV-1 sequences covering (**A**) *gag* (1,264 to 2,148), (**B**) *env* (6,450 to 8,480), (**C**) *pol* (2,253 to 3,272), and (**D**) *pol* from drug naïve individuals (2,253 to 3,272) were classified into BPANDEMIC (red branches) and BCAR (blue branches) lineages according to the topology obtained in each tree. Node support (SH-aLRT) for subtype B and BPANDEMIC monophyletic groups are indicated. The trees were rooted using HIV-1 subtype D sequences. Branch lengths are drawn to scale with the bar at the bottom indicating nucleotide substitutions per site.
