# Supplementary material for: Few amino acid signatures distinguish HIV-1 subtype B pandemic and non-pandemic strains
Source: PLoS One. 2020 Sep 22;15(9):e0238995. doi: 10.1371/journal.pone.0238995 (PMC7508567; doi:10.1371/journal.pone.0238995)
Supplement: S1 Table — The table summarizes the results of the classification of the full-length (FL) and partial HIV-1 subtype B sequences in the BPANDEMIC or BCAR based on their placement on ML phylogenetic trees displayed in Fig 1 and S1 Fig. All sub-datasets are accompanied by their sampling range. *Country codes are in accordance with ISO 3166–1. (PDF) [file pone.0238995.s002.pdf]

S1 Table. Classification of the HIV-1 subtype B sequences in the BPANDEMIC or BCAR [illegible]

Dataset	Country*	Subtype B	BPANDEMIC (%)	BCAR (%)	Putative Recombinants (%)
FL	DO	4	-	4 (100)	-
	HT	6	-	5 (83)	1 (17)
	JM	8	4 (50)	4 (50)	-
	TT	5	-	5 (100)	-
	BR	118	106 (90)	1 (1)	11 (9)
	US	330	306 (93)	9 (3)	15 (5)
	Others	35	34 (97)	-	1 (3)
	**Total**	**506**	**450 (88)**	**28 (6)**	**28 (6)**
	**Years**	1978-2015	1978-2015	1983-2011	1986-2012
gag (HXB2 coordinates 1,264 to 2,148)	DO	4	-	4 (100)	-
	HT	12	3 (25)	9 (75)	-
	JM	18	8 (44)	10 (56)	-
	TT	5	-	5 (100)	-
	BR	107	106 (99)	1 (1)	-
	US	315	306 (97)	9 (3)	-
	Others	34	34 (100)	-	-
	**Total**	**495**	**457 (92)**	**38 (8)**	-
	**Years**	1978-2015	1978-2015	1983-2011	
pol (HXB2 coordinates 2,253 to 3,272)	BS	14	5 (36)	9 (64)	-
	DO	144	39 (27)	105 (73)	-
	HT	5	-	5 (100)	
	JM	151	78 (52)	73 (48)	-
	TT	5	-	5 (100)	
	BR	107	106 (99)	1 (1)	
	US	315	306 (97)	9 (3)	
	Others	34	34 (100)	-	-
	**Total**	**775**	**568 (74)**	**207 (26)**	-
	**Years**	1978-2015	1978-2015	1983-2011	
env (HXB2 coordinates 6,450 to 8,480)	DO	5	1 (20)	4 (80)	-
	HT	11	-	11 (100)	-
	JM	9	5 (56)	4 (44)	-
	TT	40	-	40 (100)	-
	BR	107	106 (99)	1 (1)	
	US	315	306 (97)	9 (3)	
	Others	34	34 (100)	-	
	**Total**	**529**	**452 (87)**	**69 (13)**	-
	**Years**	1978-2015	1978-2015	1983-2011	
pol-drug näive (HXB2 coordinates 2,253 to 3,272)	CU	85	84 (99)	1 (1)	-
	DO	143	42 (29)	101 (71)	-
	HT	5	1 (20)	4 (80)	-
	JM	26	11 (42)	15 (58)	-
	GF	255	109 (43)	146 (57)	-
	SR	57	33 (58)	24 (42)	-
	Others	6	1 (100)	5 (100)	
	**Total**	**577**	**281 (49)**	**296 (51)**	-
	**Years**	2005-2012	2005-2012	2005-2012	

The table summarizes the results of the classification of the full-length (FL) and partial HIV-1 subtype B sequences in the BPANDEMIC or BCAR based on their placement on ML phylogenetic trees displayed in Figures 1 and S1. All sub-datasets are accompanied by their sampling range. *Country codes are in accordance with ISO 3166-1.
