# Supplementary material for: Few amino acid signatures distinguish HIV-1 subtype B pandemic and non-pandemic strains
Source: PLoS One. 2020 Sep 22;15(9):e0238995. doi: 10.1371/journal.pone.0238995 (PMC7508567; doi:10.1371/journal.pone.0238995)
Supplement: S2 Table — The table summarize the full-length (FL) HIV-1 Subtype D sequences used in our study and their sampling range. * Country codes are in accordance with ISO 3166–1. (PDF) [file pone.0238995.s003.pdf]

S2 Table. HIV-1 subtype D full-length genome sequences.

Country	*N*
CD	7
CM	5
KE	3
SN	1
UG	1
TZ	1
**Total (Years)**	18 (1983 – 2011)

The table summarize the full-length (FL) HIV-1 Subtype D sequences used in our study and their sampling range. * Country codes are in accordance with ISO 3166-1.
