# Supplementary material for: Few amino acid signatures distinguish HIV-1 subtype B pandemic and non-pandemic strains
Source: PLoS One. 2020 Sep 22;15(9):e0238995. doi: 10.1371/journal.pone.0238995 (PMC7508567; doi:10.1371/journal.pone.0238995)
Supplement: S3 Table — The table summarizes the predicted usage of chemokines receptors CCR5 and CXCR4 based on different criteria: 1) the Geno2Pheno algorithm, which classifies the sequences between R5 variants or X4 and R5X4 dual-tropic variants; 2) the 11/25 Rule, which asses the presence of arginine (R) or lysine (K) at position 11 of env V3 sequences and/or K at position 25; 3) the combination of R at position 25 of V3 and a net charge of ≥ 5. (PDF) [file pone.0238995.s004.pdf]

S3 Table. Predicted co-receptor usage by BCAR and BPANDEMIC *env* sequences.

Subtype B Lineage	Geno2Pheno		11/25 Rule		25R/Net Charge (%)
	R5-tropic (%)	X4- or R5/X4-tropic (%)	11 R/K (%)	25K (%)	
BCAR (*n* = 59)	68	32	5	3	-
BPANDEMIC (*n* = 450)	71	29	6	4	1
p-value	*0.682*		*0.914*	*1*	-

The table summarizes the predicted usage of chemokines receptors CCR5 and CXCR4 based on different criteria: 1) the Geno2Pheno algorithm, which classifies the sequences between R5 variants or X4 and R5X4 dual-tropic variants; 2) the 11/25 Rule, which asses the presence of arginine (R) or lysine (K) at position 11 of *env* V3 sequences and/or K at position 25; 3) the combination of R at position 25 of V3 and a net charge of ≥ 5.
