# Supplementary material for: Few amino acid signatures distinguish HIV-1 subtype B pandemic and non-pandemic strains
Source: PLoS One. 2020 Sep 22;15(9):e0238995. doi: 10.1371/journal.pone.0238995 (PMC7508567; doi:10.1371/journal.pone.0238995)
Supplement: S4 Table — The table summarizes the surveillance drug-resistance mutations (SDRM) identified in PR/RT BCAR and BPANDEMIC sequences retrieved from drug naïve subjects. PI (protease inhibitor), NRTI (nucleoside analog reverse-transcriptase inhibitors), NNRTI (non-nucleoside analog reverse-transcriptase inhibitor). The p-values obtained in chi-squared tests are listed in the last column. (PDF) [file pone.0238995.s005.pdf]

S4 Table. Prevalence of transmitted drug-resistance mutations in BCAR and BPANDEMIC PR/RT sequences.

Resistance Category	BCAR		BPANDEMIC		*p*-value
	# seq. analyzed	# seq. with SDRM (%)	# seq. analyzed	# seq. with SDRM (%)	
**any SDRM**	296	25 (8.4)	281	34 (12.1)	*0.190*
**PI SDRM**	295	5 (1.7)	280	6 (2.1)	*0.930*
**NRTI SDRM**	295	17 (5.8)	278	18 (6.5)	*0.856*
**NNRTI SDRM**	295	10 (3.4)	278	18 (6.5)	*0.129*

The table summarizes the surveillance drug-resistance mutations (SDRM) identified in PR/RT BCAR and BPANDEMIC sequences retrieved from drug naïve subjects. PI (protease inhibitor), NRTI (nucleoside analog reverse-transcriptase inhibitors), NNRTI (non-nucleoside analog reverse-transcriptase inhibitor). The *p*-values obtained in chi-squared tests are listed in the last column.
