# Supplementary material for: Few amino acid signatures distinguish HIV-1 subtype B pandemic and non-pandemic strains
Source: PLoS One. 2020 Sep 22;15(9):e0238995. doi: 10.1371/journal.pone.0238995 (PMC7508567; doi:10.1371/journal.pone.0238995)
Supplement: S1 File — (PDF) [file pone.0238995.s006.pdf]

FULL LENGTH GENOMES:

KJ704795	AB485638	JN944930
KJ704794	FJ496145	FJ496167
KJ704793	AF069140	DQ853436
KJ704792	DQ487190	JN944917
KJ704791	AY713410	JQ403101
KJ704789	JN024274	KT124799
AY835759	KU678161	KT124748
AY835777	AY835753	JN944911
AY835770	AY308760	FJ496151
AY835754	JN024422	FJ469738
K02007	AY331289	FJ496072
AY835779	AY331287	JQ403105
AY835762	AY331284	KT124764
AY352275	AY331282	FJ469728
M17449	KT124770	FJ469730
AB221005	KT124745	FJ469734
AY835769	KT124744	FJ469751
AY835755	AY560110	FJ469754
U69584	AY560109	FJ469757
AY835774	AY560108	FJ469758
AY835757	AY560107	FJ469741
AF004394	KT124771	FJ469737
M38429	KT124769	FJ469688
M93258	KT124747	FJ469687
AF286365	EF175209	FJ469760
AY835766	AY331296	FJ469762
89.U39362	JQ403100	KT124807
AY713412	KT124798	KT124781
AY173955	KT124776	FJ469742
AY173954	KT124759	FJ469755
AY173952	EF363122	FJ469739
FJ496158	KT124806	FJ469764

FJ469753	KT124778	JF689864
FJ469701	KF990605	JF689863
FJ469686	JF320482	JF689862
FJ469765	JN024459	JF320169
FJ469759	FJ469683	JQ403095
FJ469740	JF320361	JQ403068
FJ469743	JF320043	KT124791
FJ469770	FJ469717	JF320044
EF363127	FJ469703	JF320003
EF363124	FJ469718	JF320613
JQ403107	FJ469724	JF320036
JQ403104	FJ469682	KF990608
KT124758	JF320185	JF320120
FJ469733	JN024469	JF320097
FJ469695	FJ469747	JF320038
FJ469732	JF320059	FJ469709
JN024203	JF320054	JF320145
FJ469772	JN024450	JF320526
FJ469722	JN024339	JF320160
EU547186	FJ496000	FJ469708
FJ469697	FJ495937	JF320144
FJ469745	FJ495818	JF320356
FJ469698	JF689876	FJ469723
JN024100	JF689875	FJ469714
FJ469684	JF689874	FJ469696
DQ886037	JF689873	JF320615
FJ496081	JF689872	JF320363
JF689860	JF689871	JF320048
JF689859	JF689870	FJ469700
JF689857	JF689868	JN944897
JF689856	JF689867	JF320564
JF689854	JF689866	JF320263
JF689852	JF689865	FJ469691

KY112056	JF320460	KX228807
JF689892	JF689897	JN397364
JF689890	JF689896	KC473828
JF689889	JF689895	KC473826
JF689887	JF689894	KC473829
JF689886	JQ403099	KC473827
JF689884	JQ403091	KC473825
JF689883	JQ403087	KX228803
JF689879	JQ403083	KX228802
JF689877	JQ403069	KU677989
JQ403093	JQ403067	KU678069
JQ403071	JQ403061	KC473834
JQ403096	JQ403035	KC473835
JQ403092	JQ403088	KC473833
JQ403078	JQ403086	KC473831
JQ403066	JQ403074	KF384813
JQ403064	JQ403070	KF384812
JQ403060	JQ403030	KF384811
JQ403056	KT124793	KF384810
JX863922	KT124774	KF384809
JX863921	KT124761	KF384808
KT124801	KY112127	KF384807
JF320559	KY112429	KF384806
FJ469721	KY112136	KF384805
JF320530	KY112461	KF384804
JF320179	KY112522	KF384803
JF320150	GU733713	KF384802
JF320577	JX140657	KF384801
JF320197	KC473824	KF384800
FJ469689	KC935959	KF384799
FJ469707	KC935958	KF384798
JF320045	JX863924	KU641402
FJ469713	KX228825	KT124809

KT124775	KX505504	FJ195090
KT124766	KX505552	FJ195091
KF526174	KX505446	JF320008
KF526228	AB485641	JF320012
KF526261	AY037268	JF320017
KF526298	AY037269	JF320018
KT124790	AY037270	JF320028
KT124785	AY037282	JF320186
KT124784	AY561236	JF320189
KT124768	AY561238	JF320191
KT124756	AY561240	JF320196
KT124750	AY781126	JF320198
KY778593	AY781127	JN235958
KY778473	DQ358809	JN235959
KU901995	DQ358810	JN235965
KU901976	DQ383746	JN251901
KT124796	DQ383748	JN251906
KU749387	DQ383749	JN692431
KY766150	DQ383751	JN692432
KT284371	DQ383752	JN692433
KY778390	EF637046	JN692435
KY778345	EF637047	JN692439
KX505583	EF637048	JN692440
KX505652	EF637049	JN692443
KX505686	EF637050	JN692444
KY658693	EF637051	JN692445
KU678025	EF637053	JN692446
KY778289	EF637054	JN692450
KX505739	EF637056	JN692451
KU678049	EF637057	JN692452
KX505419	FJ195086	JN692453
KX505475	FJ195088	JN692454
KX505396	FJ195089	JN692455

JN692457	KT427685	KT427772
JN692460	KT427687	KT427781
JN692461	KT427693	KT427783
JN692462	KT427694	KT427786
JN692463	KT427699	KT427787
JN692465	KT427700	KT427791
JN692472	KT427701	KT427803
JN692473	KT427706	KT427805
JN692474	KT427709	KT427808
JN692475	KT427711	KT427815
JN692476	KT427713	KT427819
JN692479	KT427715	KT427824
JN692480	KT427717	KT427826
KJ849767	KT427718	KT427827
KJ849780	KT427723	KT427830
KJ849784	KT427729	KT427831
KJ849785	KT427730	KT427869
KJ849790	KT427742	KT427870
KJ849799	KT427744	KY658683
KJ849801	KT427745	KY658684
KJ849803	KT427748	KY658685
KJ849804	KT427751	KY658686
KJ849808	KT427753	AY586542
KJ849811	KT427754	AY586543
KJ849812	KT427755	EU839596
KJ849814	KT427757	EU839597
KJ849815	KT427763	EU839598
KJ849818	KT427764	EU839600
KT427650	KT427765	EU839601
KT427670	KT427768	EU839602
KT427675	KT427769	EU839603
KT427676	KT427770	EU839604
KT427682	KT427771	EU839605

EU839606	FJ211651
EU839607	
EU839608	
EU839609	
EU839610	
KY658702	
HM030559	
HM030560	
HM030561	
HM030562	
HM030563	
HM030564	
HM030565	
M17451	

gag

(HXB2: 1,264-2,148)

FJ211725	FJ211737
FJ211625	
FJ211613	
FJ211712	
FJ211762	
FJ211749	
FJ211676	
FJ211791	
FJ211790	
FJ211701	
FJ211805	
FJ211792	
FJ211782	
FJ211777	
FJ211638	

pol

(HXB2: 2,253-3,272)

DQ518437	JN713693	JN713601
DQ518434	GU437891	JN713630
DQ518438	JN713588	JN713571
JN713587	JN713661	JN713680
GU437882	JN713568	JN713657
GU972720	GU972771	JN713599
JN713702	JN713669	JN713602
JN713684	JQ237877	JN713616
JN713665	JQ237874	JN713629
JN713631	GU972750	GU437893
JN713652	GU437904	GU972728
JN713706	JQ237891	GU972722
JN713585	GU972739	GU437912
JN713576	GU437906	GU437900
DQ518431	JN713597	GU437880
DQ518427	JN713683	GU437903
DQ518430	JN713604	GU437907
GU437913	GU972781	JQ237889
JN713582	GU972727	JQ237880
JN713677	GU972766	JQ237883
JN713591	JN713697	JQ237875
JN713707	JN713624	JQ237870
JN713615	JN713633	JQ237882
JN713569	JN713653	JQ237879
JN713610	DQ518429	JQ237864
JN713673	JN713681	GU972747
JN713612	JN713709	GU972782
JN713594	JN713590	GU972776
JN713666	JN713617	GU972738

GU972779	JN713647	JN713577
GU972742	JN713690	JN713613
GU972762	JN713692	JN713622
GU972760	JN713621	JN713578
GU972753	JN713640	JN713668
GU972735	JN713611	JN713570
GU972746	JN713626	JN713695
JQ237872	JN713605	JN713598
JQ237887	JN713656	GU972774
GU972725	JN713685	JN713572
GU972724	JN713646	GU972723
JQ237869	JN713655	GU972777
GU437887	JN713642	JQ237888
JQ237890	JN713596	JN713592
GU972769	JN713678	JN713676
GU437884	JN713608	JN713696
JQ237863	JN713688	JN713583
GU972783	JN713595	JN713638
GU437897	JN713708	JN713687
JQ237855	JN713664	JN713654
JQ237852	JN713600	JN713644
GU437894	JN713662	JN713574
GU437911	JN713689	JN713703
GU972748	JN713675	JN713670
GU972780	JN713579	JN713567
JQ237856	JN713632	JN713620
JQ237860	JN713573	DQ518440
JQ237871	JN713701	JN713658
GU972765	JN713643	JN713580
DQ518435	JN713635	JN713589
JN713674	DQ518432	JQ237857
JQ237867	JN713607	DQ518436
JN713649	JN713705	DQ518433

GU972756	GU437879	JN713679
GU972754	GU972775	JN713645
JN713636	GU972772	JN713650
JN713691	GU437877	JN713593
JN713623	JN713628	JN713606
GU437889	JN713651	JN713614
GU437886	JN713686	JN713586
JN713698	GU437878	JN713682
GU437902	GU972733	GU437898
GU437892	GU972737	JN713660
GU972734	GU972752	JQ237885
GU972732	GU972740	JQ237868
GU972730	GU972736	JQ237876
GU972761	GU972741	JQ237858
GU972751	GU972764	JQ237881
GU972759	JN713639	JQ237878
GU972767	JN713581	GU972743
GU972719	JN713699	JQ237865
JQ237886	JN713671	JQ237859
GU437896	JN713694	JN713641
GU972721	JN713619	GU972744
GU437909	GU972757	JQ237884
GU972763	JN713672	DQ518439
GU437888	JN713700	JN713575
GU437881	JQ237866	JN713603
GU972749	JQ237853	JN713648
GU972768	JN713625	JN713618
GU972770	JN713663	GU972729
JQ237851	JN713609	DQ518428
GU437895	JN713704	GU972773
GU972731	GU437883	JQ237873
GU437885	GU972726	JN713627
GU437899	GU972758	GU437901

GU437890	AF277054
JN713667	EF593264
GU972755	EF593268
GU437908	EF593266
JQ237862	EF593265
GU437910	AF277062
GU437905	AF277072
JQ237854	AF277063
JN713584	AF277057
GU972778	AF277060
GU972745	AF277056
JQ237861	

env

(HXB2: 6,450-8,480)

JF680925	AF277068
FJ817365	AF277067
EF593240	HQ217651
AF277059	AF277055
AF277066	AF277058
EF593267	EF593261
HQ217760	AF277065
EF593260	AF277071
AF277073	AF277064
AF277070	AY669724
AF277069	U08446
EF593262	U08447
AF277074	U08443
AF277061	U08441
AF277075	AY669721
EF593263	
EF593259	

***pol* (Drug Naïve)**

(HXB2: 2,253-3,272)

KT998079	KT998221	KT998148
KT998159	KX390957	JN713698
KT998231	KT998040	JN713691
KX390962	KT998083	JN713623
JX194204	KT998225	KT998262
KT998074	KP688156	KT998140
JN713660	GU437890	KT998117
HQ655409	JN713667	JN713580
JN713639	JN713671	DQ832138
KT998243	JN000037	HM030561
KT998276	KP688151	JN713694
KT998096	JN713641	JN713619
JN713658	KT998177	KP688135
KT998245	KT998078	GU972741
KT998280	KT998205	KT998199
KT998003	KT998008	JN713618
JN713603	KU052749	KT998213
GU437905	KU052750	KT998010
JN713575	KT998157	JN713663
KX390969	KT998214	HQ655399
KX390881	KT998013	KT998023
KT998278	KT998049	KT998259
GU437898	JN713651	KT998250
KT998084	KP688154	JN713584
KT998227	HM030565	JN713682
KT998047	GU972734	JN713686
KX390963	GU972721	JN713586
KX390908	HM030563	KT998106
KT998196	GU972758	KT998229

KT998260	KP688174	KT998258
JN000008	KP688114	JN713593
JN000048	KP688157	KT998087
JN000005	HQ655381	JN713606
JN000016	KP688110	KP688140
JX194216	HQ655360	JN713700
HQ655396	JN000050	KX390940
JN000033	HQ655375	JX194186
KP688158	HQ108365	KX390894
KP688155	HQ108355	KP688130
JX194235	JX194183	JN713628
HQ655377	JX194181	JN713614
HQ655356	JX194189	KP688134
JX194226	JX194194	HQ655383
HQ655379	HQ655372	JX194214
JN000041	KP688106	HQ655380
HQ655351	KP688152	JN000061
HQ655394	JN713696	KT998066
HQ655393	JN713583	KT998024
JN000038	KP688084	KT998247
KP688138	JX194198	KX390943
HQ655352	JN713672	KX390885
JX194224	KT998042	KT998035
HQ655350	GU972752	KX390914
JX194201	JN713650	KT998146
JX194193	KU052748	KT998101
HQ655355	JN713679	KX390878
KP688160	JN713636	KX390956
JX194218	JX194202	KX390948
KP688087	KP688099	KX390973
KP688163	JN713589	KX390970
HQ108359	KP688164	KX390892
KP688145	JN000009	KT998016

KX390901	JX194195	KT998285
KT998282	HQ655401	KT998004
KX390898	HQ655345	KX390939
KT998065	JX194200	KX390947
KT998180	JN713704	KX390950
KT998169	JN713648	KX390976
KT998175	KT998027	KT998020
KX390961	KT998036	KT998115
KT998188	KP688100	KT998099
KT998232	KT998252	JN713625
KT998184	KT998107	KT998131
KX390933	KT998017	HQ655390
KT998219	KX390975	KT998183
KT998257	KP688097	KT998202
KX390942	KP688147	JX194209
KT998011	KT998191	JN000025
KT998238	KT998237	HQ655387
KT998239	KT998244	KT998103
KT998026	KT998110	KT998018
KT998193	KT998166	JN713680
KT998201	KT998273	JN713657
KT998071	KT998217	JN713602
KT998198	KT998025	JN713599
KT998272	KX390882	KX390972
KT998268	KX390886	KT998037
KT998181	KX390941	JN713645
KT998046	KX390968	KT998207
KT998161	KX390937	JN713581
KT998189	KT998122	KU052744
KT998211	KT998095	KT998123
KP688165	KT998060	KT998283
KT998263	KT998072	KT998266
JX194196	KT998053	KT998039

KX390958	KT998209	KT998248
KT998045	KT998081	KT998086
KT998055	KT998251	EU839601
KT998163	KT998030	JN713591
KT998100	KT998149	JN713677
KT998048	KT998241	JN713707
KX390954	KT998271	JN713615
KX390967	KT998255	JN713610
KT998062	KT998261	JN713569
KT998118	KT998160	KT998130
KT998265	EU839607	JN713582
KT998033	KT998279	KT998009
KU052757	KT998185	JN713706
KT998151	KT998186	JN713585
KT998109	EU839606	JN713576
KT998179	KU052745	EU839600
KT998156	JN713597	JN713652
KT998031	EU839608	KT998128
KT998080	JN713616	KT998098
KT998270	EU839610	KT998050
KT998145	EU839609	EU839603
KX390888	JN713568	GU972771
JN713653	KU052747	JN713697
KT998076	EU839602	KT998019
KU052754	JN713683	KT998210
KT998112	KT998206	KT998192
KT998218	KU052755	JN713673
KT998051	JN713604	KU052743
KT998208	KX390879	KT998092
KT998021	KT998111	EU839596
KT998052	KT998264	KT998212
KT998028	HM030564	KT998022
KT998178	KT998164	JN713669

JN713681	JN713702	KT998005
KT998056	KT998038	KT998176
KT998204	KT998057	KT998012
KX390952	KT998090	KT998197
KT998105	KT998002	KT998277
KT998034	KX390955	KT998015
KX390936	KT998094	KT998228
KT998154	KT998215	KT998147
KX390910	KT998195	EU839604
KT998104	KT998224	JN713649
KT998119	KU052742	JN713647
KT998235	KT998014	JN713690
KX390949	JN713684	JN713621
KX390977	JN713665	JN713692
KT998254	JN713631	JN713640
KX390880	KT998226	JN713611
KT998139	JN713633	JN713626
KX390945	JN713624	JN713605
KX390965	EU839605	JN713656
KT998286	GU972782	JN713685
KT998043	HM030560	JN713646
KT998182	KT998234	JN713655
KT998274	GU972780	JN713642
KT998236	GU972753	JN713596
KT998284	GU972765	JN713678
KX390944	HM030559	JN713608
KX390889	GU972720	JN713688
KX390966	KT998097	JN713595
KT998200	JN713587	JN713708
KX390964	JN713592	JN713664
KT998108	JN713709	JN713600
KT998113	KT998069	JN713662
KT998162	KT998070	JN713675

JN713689
JN713579
JN713573
JN713632
JN713701
JN713643
JN713635
JN713705
JN713607
KT998124
KT998082
KT998085
KT998253
KT998063
KT998054
JN713668
JN713577
JN713578
JN713622
KT998275
JN713613
JN713570
JN713695
KT998230
KT998059
JN713598
JN713676
JN713572
KT998058
KT998068
GU972774
GU972777
KU052753
JN713687
JN713654
JN713638
JN713644
EU839597
JN713567
JN713670
JN713574
JN713703
JN713620
JN713661
KT998067
KT998089
JN713612
JN713594
JN713693
JN713666
JN713629
KT998129
KT998126
KX390971
KT998269
KT998223
JN713674
HQ108363
KT998249
KT998152
KT998116
KT998203
KT998032
KT998155
KT998222
KX390884
KX390918
KX390897
JN713609
KU052752
EU839598
KU052758
KX390916
KT998216
JN713699
KT998256
JN713601
JN713630
JN713571
KT998064
KU052751
KT998007
JN713590
JN713617
GU972727
GU972766
GU972781
KU052746
JN713588
JN713627
KT998041
KX390960
KT998233
KT998242
